# Associated-Factor Studies in Biomedical Research: Foundations, Limitations, and Criteria for Appropriate Use

**DOI:** 10.3390/epidemiologia7050119

**Published:** 2026-08-22

**Authors:** Víctor Juan Vera-Ponce, Jhosmer Ballena-Caicedo

**Affiliations:** Facultad de Medicina (FAMED), Universidad Nacional Toribio Rodríguez de Mendoza de Amazonas (UNTRM), Chachapoyas 01001, Amazonas, Peru; 7330178022@untrm.edu.pe

**Keywords:** associated factors, Table 2 fallacy, events per variable, exploratory models, causal inference, observational studies

## Abstract

Associated-factor studies constitute a substantial portion of the observational epidemiology literature. However, their proliferation has raised important methodological concerns. Many are published without a clear research question, repeat well-established associations, or use causal language to interpret coefficients from exploratory models. This narrative methodological review and critical reflection provides guidance for determining when associated-factor studies add genuine scientific value, how to plan sample size using events per variable, and how to interpret and report results appropriately. We review the nature of exploratory models and how they differ from predictive and causal models. We examine the Table 2 fallacy and its implications for interpreting adjusted coefficients. We discuss the events per variable criterion as a framework for sample-size planning in multivariable studies, present illustrative examples of both appropriate and inappropriate uses of these analyses, and provide an operational decision and minimum-reporting checklist for authors, reviewers, and editors. Associated-factor studies have a legitimate role when the phenomenon is poorly understood, when there is a genuine evidence gap in a truly different population, or when the explicit aim is hypothesis generation rather than causal inference. Their scientific contribution is limited when they merely confirm well-established associations, use causal language without a causal design, or are conducted simply because data are available.

## 1. Introduction

In observational biomedical research, it is common to find studies that, under the label of “associated factors,” collect multiple variables, fit a multivariable model, and report statistically significant associations with a health outcome. This approach is widespread in clinical and epidemiologic journals and can be useful when the goal is genuinely exploratory. However, the proliferation of these studies in topics where the risk-factor profile is already well established has raised methodological questions about their actual contribution to the body of knowledge [1,2,3].

The core problem is that many “associated factors” studies do not clearly state their research question: is the aim to describe how an outcome is distributed according to population characteristics, to predict the outcome in new individuals, or to estimate the causal effect of a specific exposure? These three objectives require different analysis strategies, adjustments, and interpretations. The failure to distinguish among these purposes is a central concern in modern epidemiologic literature [1,4,5,6].

This confusion has practical consequences. In well-studied topics, an additional analysis that merely confirms known associations may contribute little. Moreover, when expressions suggesting causality (“increased risk,” “protective factor”) are used to present results from an exploratory model, both the scientific interpretation and the potential application of findings are compromised [3,7,8]. Additionally, the lack of adequate sample-size planning, often missing in multivariable studies, can lead to unstable, overfitted models with overestimated associations [9,10].

This article is a narrative methodological review and critical reflection, not a systematic review. The cited literature was selected purposively to synthesize major methodological arguments on descriptive and exploratory regression, the Table 2 fallacy, sample-size planning, prediction modeling, causal inference, and reporting practice. The article therefore should not be read as a comprehensive evidence synthesis, but as an integrative methodological guide for evaluating a common biomedical publication format.

Its contribution is operational: it translates dispersed methodological debates into practical criteria for deciding when an associated-factor study is justified, how it should be framed, and how its results should be reported. In doing so, it addresses a recurrent gap in biomedical publishing: many manuscripts use the label “associated factors” without specifying whether the underlying purpose is descriptive, exploratory, predictive, or causal.

## 2. The Nature of Exploratory Models

In this article, “associated-factor studies” refers to observational analyses in which a health outcome is modeled as a function of multiple candidate covariates and the resulting adjusted coefficients are reported as “factors associated with” the outcome. This category overlaps with exploratory multivariable regression and adjusted descriptive epidemiology, but it is distinct from formal prediction modeling, which requires performance assessment and validation, and from causal-effect estimation, which requires a prespecified exposure, target estimand, and causal identification strategy. The term is therefore used here as a practical label for a common biomedical publication format, not as a separate statistical model class.

Multivariable statistical models can be used for three distinct purposes: describing/exploring patterns of association, predicting outcomes, or estimating causal effects. Although the mathematical specification of a logistic or Cox regression may be the same regardless of the purpose, the underlying logic, the choice and interpretation of covariates, and the criteria for judging the quality of results differ substantially among these three goals [1,6].

In an exploratory model, a specific causal hypothesis is generally not posited, nor is the goal to build a clinical prediction tool. The focus is on identifying which variables show an association with the outcome once adjusted for other covariates. This “simultaneous adjustment” is a form of mutual statistical control: the coefficient for each variable describes its conditional association with the outcome given the other variables in the model [6].

This description, although useful for summarizing information, conceals several methodological tensions. The first is that simultaneous adjustment does not necessarily constitute appropriate confounding control for each variable. As Westreich and Greenland [11] argued, the optimal adjustment set depends on the causal structure of the specific question. In a model that includes multiple variables without a defined causal question, some covariates may act as confounders for certain variables but as mediators or colliders for others. Therefore, interpreting the adjusted coefficient for a given variable may be misleading, even with the correct *p*-value and confidence interval [7,8,11].

The second tension is that results from exploratory models are often narrated using language that suggests causality. Expressions such as “increased risk” or “was protective” can be read as cause–effect relationships even when the design does not allow such conclusions. This tendency has been documented in multiple disciplines and constitutes one of the most common interpretive errors in the associated-factors literature [3,12].

The third tension is that the scientific value of an exploratory analysis depends critically on context and the state of knowledge. When a phenomenon is emerging and uncertainty is high, these analyses can provide early signals about what characteristics are associated with the outcome. For example, in the early stages of the COVID-19 pandemic, exploratory multivariable analyses in hospitalized cohorts were valuable for identifying associations with severe disease and mortality [10,13]. In contrast, when the risk profile is already well established (e.g., type 2 diabetes mellitus), adding another analysis that confirms age, BMI, and physical inactivity as “factors associated” with diabetes in a new sample may contribute very little, especially if the population does not differ meaningfully from those already studied [14]. The distinction between the fitted regression coefficient, the scientific estimand, the recommended interpretation, and the corresponding reporting language is summarized in Box 1.

Box 1 **From fitted coefficient to scientific estimand.**

In an exploratory model such as g{E(Y|X,Z)} = beta0 + beta1X + beta2Z, the coefficient beta1 represents a model-conditional association between X and Y, conditional on the variables included in Z and on the model specification. It should not be interpreted automatically as a causal contrast such as E(Y if X = 1)–E(Y if X = 0) or as a causal risk ratio. A causal interpretation would require an explicit target estimand and assumptions such as exchangeability, positivity, consistency, correct temporal ordering, and appropriate control of confounding. Without those elements, the coefficient remains an adjusted association within the fitted model.

## 3. The Table 2 Fallacy: Origins and Consequences

In 2013, Daniel Westreich and Sander Greenland published in the American Journal of Epidemiology the article that popularized the term “Table 2 fallacy” to describe a common error in reporting multivariable models: presenting all adjusted coefficients in a single table and interpreting each one as if it were an independent, comparable “effect” of its respective variable. According to the authors, this practice promotes an unjustified equivalence in interpretation among coefficients that, in fact, may have very different causal meanings depending on their role in the causal structure [11].

To understand why this is problematic, it is helpful to recall what “adjusting” means in a regression model. In a model (e.g., logistic regression), the coefficient for a variable is interpreted as an association conditional on the other variables included. However, the variables in the model may play different roles in the causal graph: some may be confounders for a particular variable (and it is correct to adjust for them), but others may be mediators (adjusting for which blocks the total effect) or colliders (adjusting for which can create spurious associations). In a single model with multiple variables, the same adjustment set is applied to all of them. It is not possible to “choose” different adjustment sets for different variables within the same model [3,7,8,11].

The problem arises because different variables within the same model may occupy different causal roles. For example, age could be a confounder for the smoking–mortality relationship, but smoking may act as a mediator for the alcohol–mortality relationship if part of the effect of alcohol on mortality is mediated through smoking habits. Adjusting for smoking is correct in the first case, but can be incorrect in the second, as it could block a relevant causal pathway. The Table 2 fallacy occurs when these differences are ignored, and all coefficients are presented and interpreted as if they were “independent effects” and “risk factors” with comparable meaning [3,11].

A single model that includes all variables simultaneously cannot ensure the “best adjustment” for each coefficient if the reader interprets them as causal effects. What it provides is mutual adjustment with a clear statistical meaning (conditional associations), but its causal interpretation varies from coefficient to coefficient and requires external analysis (e.g., a directed acyclic graph, DAG) that most associated-factor studies do not include [7,8].

The consequences of the Table 2 fallacy are several. First, it promotes unjustified causal interpretations (“independent risk factor,” “increased risk,” “was protective”) that can turn conditional associations into implicit recommendations for interventions. Second, it creates a false sense of completeness: the reader may believe that the table shows “all the important factors and their effects,” when in fact the results are critically dependent on which variables are included and what role they play in the causal structure. Third, it can mask important problems such as confounding by indication, over-adjustment, or collider bias, which are not detectable simply by looking at coefficients and *p*-values [7,11,15].

It is important to note that Westreich and Greenland do not argue that multivariable models are “useless” or that reporting multiple coefficients is prohibited. Their argument is that authors and readers must be careful in interpreting each coefficient and that the presentation of multiple adjusted coefficients in a single table should be accompanied by an explicit discussion of the causal structure and the limitations of that joint interpretation [11]. A scoping review found that the Table 2 fallacy is prevalent even in oral-health research, suggesting that this error is not confined to general epidemiology [12].

Two additional issues further complicate interpretation of “Table 2” coefficients. First, in logistic regression, adjusted odds ratios may differ from crude odds ratios because of non-collapsibility, even in the absence of confounding [16]. Therefore, a change in the odds ratio after adjustment should not automatically be interpreted as evidence that confounding has been removed or that an “independent effect” has been isolated. Second, a single adjusted coefficient may obscure effect modification or nonlinear associations. If the association varies across clinically relevant subgroups or across the range of a continuous predictor, a single main-effect coefficient can be an inadequate summary. Thus, the Table 2 fallacy is not only a problem of confounders, mediators, and colliders; it is also a problem of whether the model structure matches the scientific question.

## 4. Sample Size: Events per Variable

Sample-size calculation for associated-factor studies poses an important challenge. Traditional sample-size formulas used in observational studies often focus on detecting a difference between two groups or estimating the strength of association of a single exposure, using parameters such as effect size, significance level, power, and expected prevalence. These formulas are adequate when the study has a single main exposure and a pre-established analysis plan [17,18].

When the goal is an exploratory multivariable analysis, these traditional formulas are generally inappropriate because they were not designed for scenarios in which multiple variables are examined simultaneously. The issue is not only detecting specific effects but also model stability: ensuring that the estimated coefficients are reliable and not excessively influenced by chance given the number of parameters being estimated [17,18,19].

A practical way to approach this issue is the concept of events per variable (EPV), more precisely understood as the ratio between the amount of outcome information and the number of model parameters being estimated. For binary outcomes, a useful expression is EPV = min(number of events, number of non-events)/q, where q is the number of estimated parameters or effective degrees of freedom in the model. Therefore, EPV should not be calculated only from the number of named variables. Categorical predictors with multiple levels, spline terms, nonlinear transformations, and interaction terms all increase q. EPV should be interpreted as a rough indicator of model stability and overfitting risk, not as a substitute for formal power, precision, or model-specific sample-size calculations.

The traditional, widely cited rule suggests a minimum EPV of approximately 20 for logistic regression and Cox models, based on simulation studies that observed greater bias, poorer confidence-interval coverage, and instability of coefficients at very low EPVs. However, this rule is not a universal constant: it emerged as a general guide and has been subject to debate and refinement in subsequent literature [18,19,20,21].

Subsequent research has shown that problems associated with low EPV also depend on other factors (total sample size, event fraction, correlation among predictors, prevalence of categorical predictors, and the presence of strong or very weak effects in the model). Some simulation studies have argued that the “10 EPV” rule is neither sufficient nor always necessary: in some scenarios, more than 10 EPV is needed, while in others a lower ratio may be acceptable without severe consequences [17,18,22,23,24].

In practical terms, low EPV makes automatic variable selection (e.g., stepwise procedures) particularly problematic, as such methods tend to increase instability and model optimism. In this context, it is preferable to reduce the number of candidate variables to those with the strongest prior justification and clinical plausibility, rather than including all available variables. If EPV is low, options include reducing the number of parameters, grouping categories, or accepting that the analysis is preliminary and should be replicated [17,21,24].

For investigators planning an associated-factor study, the expected EPV should be estimated before data collection using the expected number of events and non-events and the intended number of parameters or effective degrees of freedom. EPV is only one pathway for judging sample-size adequacy; it should be supplemented with model-specific criteria, including total sample size, event fraction, predictor distributions, the number of parameters, and the maximum acceptable overfitting [17,18,19,24]. When the number of events is limited relative to model complexity, penalized regression or Bayesian models with weakly informative priors may reduce instability and separation problems [25]. These approaches should be viewed as regularization strategies, not as substitutes for adequate sample size, transparent model specification, or cautious interpretation.

## 5. The Legitimate Role of Exploratory Models: Utility and Correct Interpretation

A recurring criticism—arising from the debate on the Table 2 fallacy—argues that if each coefficient requires a specific adjustment set to be interpreted causally, then reporting multiple “adjusted” coefficients from a single model is inherently misleading. This critique is technically correct when applied to causal claims. However, exploratory models serve purposes that do not require a causal interpretation for each coefficient, and in those contexts they can have legitimate and valuable uses [1,6,26].

First, exploratory models can have descriptive value: they help characterize, within a given population and context, how an outcome is distributed across auxiliary variables (e.g., sociodemographic or clinical factors) while accounting for their mutual correlation. This is especially useful in early descriptions of emerging phenomena, where descriptive analyses that account for the covariance structure can be informative even without a causal claim [6,10,13].

Second, even though they cannot establish causality, exploratory results can guide hypothesis prioritization and the design of subsequent studies: for example, a signal that persists across different adjustments or specifications may be worth investigating with a causal design (e.g., target trial emulation) [26,27]. This function of “hypothesis generation” is one of the most valued and least controversial roles of associated-factor studies, provided the generated hypotheses are not confused with conclusions in the same study [26].

Third, exploratory models can provide preliminary inputs for developing prediction models (initial candidate selection, operational variable definitions), but formal prediction model development requires performance assessment, internal/external validation, and adherence to reporting standards (e.g., TRIPOD+AI) [5,28].

Fourth, in practice, much of the clinical and epidemiologic literature reports multiple adjusted associations from a single model. In that setting, the methodological discussion should not focus solely on “prohibiting” multivariable tables, but on promoting transparent reporting practices that describe what the coefficients are (conditional associations in a specific model) and what they are not (controlled causal effects) [3,11,12].

The distinction between legitimate and problematic uses of exploratory models lies primarily in the language used to describe results. Table 1 provides examples of phrases that imply causality (and should be avoided) alongside appropriate alternatives that correctly describe exploratory associations.

Consistently applying this descriptive language has important consequences for interpreting results. When a researcher writes “treatment adherence was associated with a lower probability of poor glycemic control in the adjusted model,” the reader understands that this is a conditional association, that other factors may explain this relationship, and that additional evidence is needed before recommending specific interventions. When the same result is presented as “treatment adherence was a protective factor against poor glycemic control,” the reader may incorrectly assume that the relationship has been causally established [3,11].

This distinction has practical implications for communication with decision-makers. A clinician or policy-maker who reads “low education is a risk factor for poor glycemic control” might conclude that educational interventions will directly improve glycemic outcomes, when in reality the association may be mediated by unmeasured variables such as health literacy, economic resources, or continuity of care [29]. Transparent language avoids premature recommendations and invites deeper investigation.

In summary, exploratory associated-factor models have a legitimate place in epidemiologic research when three conditions are met: first, the topic is not saturated and there is justification to expect that the analysis will generate new information; second, results are reported transparently as model-conditional associations, without causal language; and third, sample-size planning or EPV evaluation supports sufficient model stability for the intended exploratory purpose.

## 6. When Associated-Factor Studies Add Value

Despite the limitations described, associated-factor studies can have a legitimate role in epidemiologic research when their objective is framed modestly (exploratory or descriptive) and coefficients are not interpreted as causal effects. Several scenarios can be identified in which these analyses can make a meaningful contribution [6,10,26].

First scenario: a new or emerging phenomenon. When a new disease, syndrome, or complication arises, there is urgency to identify which characteristics are associated with severe outcomes to guide clinical and public health response. Early studies on COVID-19 [10,13] illustrated this scenario: in an environment of high uncertainty, exploratory analyses with available data provided rapid and useful initial signals about factors associated with severity, mortality, and hospitalization [10,13]. In these situations, the exploratory nature of the results is usually acknowledged, and the value lies in identifying priorities for future investigation.

Second scenario: a genuinely different population with reasons to expect heterogeneity. A study may add new information if the population differs meaningfully from those previously studied (e.g., major differences in environmental exposure, ethnic composition, access to care, or disease prevalence). The key question is whether there is a priori justification to expect that associations may differ. This aligns with frameworks of generalizability and transportability, which recognize that associations can vary across populations due to structural and contextual factors [30,31].

Replication can also be scientifically valuable when it explicitly addresses reproducibility, external validity, or transportability in populations historically underrepresented in prior evidence. However, the justification should be substantive rather than merely geographic: authors should explain which contextual, biological, clinical, environmental, or health-system features could plausibly modify the observed associations or affect their interpretation.

A third scenario is when there is a novel exposure whose relationship with health outcomes has not been studied. If a new type of environmental contaminant, a new pattern of technology use, or a new dietary compound is identified, exploratory analyses are justified to generate initial evidence about potential associations. These studies follow the logic of environment-wide association studies, where the novelty lies in the exposure, not the outcome [32].

Fourth scenario: local descriptive characterization without a causal claim. A hospital or health system may benefit from describing which characteristics of its population are associated with worse outcomes for purposes of operational planning (e.g., allocating resources, identifying priority subgroups), without claiming to identify causes. These analyses have immediate operational value and do not require causal language.

What distinguishes these scenarios from problematic use is that in each case there is (a) a clear question, even if exploratory; (b) a justification for why the analysis could add new information; and (c) an explicit intention to interpret results as conditional associations, not as causal effects.

## 7. When Associated-Factor Studies Have Limited Scientific Value

There is a category of associated-factor studies that, while technically correct, have marginal scientific value because they repeat what is already known without clarifying relevant uncertainty or generating new hypotheses. Recognizing these situations is important for optimizing resources and prioritizing analyses that can advance knowledge [3,26,33].

A paradigmatic case is that of extensively investigated conditions. In type 2 diabetes mellitus, for example, there is a very large body of synthesized evidence: umbrella reviews have mapped numerous exposures and meta-analyses show consistent associations for factors such as overweight/obesity, physical inactivity, diet, and family history [14]. In this context, a new cross-sectional study that merely confirms that BMI and physical activity are “associated” with diabetes in a particular sample has limited scientific value. The finding is expected, already quantified, and does not resolve any relevant uncertainty.

A common argument used to justify these studies is “there are no studies in my country” or “there are no studies in my hospital.” This justification is insufficient unless accompanied by an explicit reason to expect true heterogeneity or to address reproducibility, external validity, or target validity. For the same reason, replicating an analysis in a new location for a well-established outcome without a specific hypothesis about why results might differ is usually a limited contribution. Recent epidemiologic frameworks on transportability and target validity help formalize this criterion: if the conditions for transporting estimates from previous studies to a new population are met, additional data collection may not be needed; when those conditions are not met, the study may be justified [30,31,34].

Another problematic scenario arises when an associated-factor study is used as a substitute for a causal or interventional question. If the real interest is “what would happen if we intervened on X?”, a cross-sectional analysis of associations cannot answer that question. In that case, the study creates an illusion of evidence without resolving the underlying question. This distinction has been formalized through frameworks such as the target trial emulation paradigm, which translates causal questions into a design closer to a hypothetical trial, even from observational data, explicitly distinguishing between predictive, descriptive, and causal goals [35].

Finally, these studies have limited scientific contribution when they are conducted primarily because “the data are available,” and multiple variables, subgroups, and models are explored until “significant” associations are obtained, without a prior analysis plan. This approach, sometimes labeled “data dredging,” generates inflated associations, false positives, and conclusions that are not reproducible [36].

## 8. Distinction from Predictive and Causal Models

To decide when an associated-factor study is appropriate and when it is not, it is useful to distinguish it from the other two main uses of multivariable models: prediction and causality. Although all three may use similar statistical models (e.g., logistic regression), the differences in objective, variable selection, interpretation, and evaluation criteria are substantial [1,6].

Prediction models. They aim to estimate the risk of an outcome in specific individuals over a defined time horizon using predictors available at the time of prediction. Their typical question is: “what is the risk that this patient will develop event Y in the next Z years, given their current profile?” In these models, all variables that improve prediction are useful, regardless of their causal role. It does not matter whether a variable is a confounder, mediator, or collider; what matters is its predictive ability. The evaluation criteria are discrimination (AUC), calibration, and external validation, rather than the “significance” of individual coefficients. To report these models, guidelines such as TRIPOD+AI are available [5,28,37].

Causal models. They aim to estimate the effect of a specific exposure or intervention on an outcome: “how much would the risk change if we modified this exposure?” In these analyses, interpretive focus is on the effect of that exposure (not all variables in the model); covariate selection is guided by a causal model (DAG) to achieve appropriate confounding control; and mediators, colliders, or instruments should not be adjusted for without explicit justification. An estimate has a causal interpretation only when the design, analysis, and assumptions allow it [3,7,8,35,38].

Exploratory associated-factor models. They typically occupy an “intermediate zone” in which the goal is neither (i) to build a prediction tool ready for use in new individuals (without formal performance assessment/validation) nor (ii) to make specific causal claims about an exposure’s effect. These models have descriptive or hypothesis-generating value, but their validity depends on their being reported as such, with appropriate language, adequate model stability, and acknowledgment that each coefficient has an interpretation conditional on all other variables in the model.

This confusion has practical consequences. When a researcher wants to know whether an exposure causes a disease, an associated-factor study cannot answer that question. When the goal is to develop a tool to identify high-risk patients, an associated-factor study is not a prediction model. And when the goal is to describe what is already known in a similar population, the contribution may be minimal. Clearly identifying the purpose of the analysis and choosing the appropriate tool for that purpose constitutes the most important methodological recommendation for this type of research [1,6,26].

## 9. Illustrative Example: Appropriate Use of an Exploratory Model

To illustrate when an associated-factor study is justified, consider the following hypothetical scenario. In 2024, a new neurologic complication was reported in patients with an emerging vector-borne tropical disease. The disease is recent, there is no prior literature on risk factors for this complication, and there is clinical urgency to identify which characteristics may help anticipate severe cases. This context represents a clear evidence gap, justifying an exploratory analysis.

A research team collects data from 380 consecutive hospitalized patients with the disease, of whom 45 develop the neurologic complication. The sample size is modest and supports only a limited exploratory model. The EPV is 45 events/5 parameters = 9, which should be considered borderline rather than definitively adequate. Therefore, the analysis should be interpreted as preliminary. Internal validation, bootstrap assessment of coefficient stability, penalized regression, or particularly cautious reporting of uncertainty should be considered, especially if predictors are correlated or unevenly distributed.

Investigators select variables based on biological plausibility and data availability: age, sex, viral load on admission, prior neurologic comorbidity, and days since symptom onset. These variables are included because they are clinically plausible, available at admission or early during hospitalization, and limited in number to preserve model stability. No automatic variable-selection procedure is used.

Table 2 presents the hypothetical results of this analysis. Investigators report crude and adjusted prevalence ratios (PRs) with 95% confidence intervals.

The appropriate interpretation of these results is explicitly exploratory. Investigators may conclude that, in this sample, high viral load on admission and the presence of prior neurologic comorbidities showed the strongest associations with the neurologic complication. These findings generate specific hypotheses that should be confirmed in larger, prospective studies with a defined causal framework. The language avoids terms such as “risk factor,” “protective effect,” or “determinant,” and instead reports “associations observed in the adjusted model” as a description of conditional patterns.

## 10. Illustrative Example: Inappropriate Use of an Exploratory Model

Contrast the previous example with a scenario in which an “associated factors” study is methodologically feasible but conceptually weak. A researcher accesses records from 1200 patients with type 2 diabetes seen at a public hospital and decides to analyze “factors associated with poor glycemic control (HbA1c > 7%).” The analysis includes 12 variables (age, sex, BMI, education, insurance type, diabetes duration, treatment adherence, comorbidities, physical activity, diet, systolic blood pressure, and number of clinical visits). The model identifies adherence, diabetes duration, and BMI as “significantly associated” with poor control, and the author concludes that these are “independent risk factors” requiring targeted interventions.

First problem: redundancy and topic saturation. In type 2 diabetes, extensive evidence shows that disease duration, health-care access/use, adherence, comorbidities, and social determinants are related to clinical outcomes. The associations found in this study are not unexpected and confirm what is already known from decades of synthesized evidence [14,29]. There is no explicit justification for why this specific hospital population would produce different results.

Second problem: mixing objectives (describing, explaining causally, or predicting). Does the researcher want to describe what characteristics patients with poor control have? That does not require a multivariable model. Does the researcher want to know whether improving adherence will improve control? That requires a causal design (e.g., a randomized trial or target trial emulation [35]). Does the researcher want to identify patients at high risk of poor control for active follow-up? That requires a prediction model with performance assessment and validation [5,28]. The “associated factors” study does not clearly answer any of these questions.

Third problem: causal interpretation not supported by the design. Suppose the analysis finds that treatment adherence is strongly associated with better glycemic control (adjusted PR = 0.35 for poor control among adherent patients). The researcher concludes: “treatment adherence is a protective factor; strengthening adherence strategies is recommended.” However, this interpretation commits several errors: (a) the association may be confounded by variables not measured (health literacy, motivation, access to medications); (b) adherence may be a marker, not a cause, of better control (confounding by indication: patients with milder disease may be more adherent because treatment is simpler [15]); and (c) the model simultaneously adjusts for variables that may be mediators or colliders (e.g., number of visits could be a mediator between insurance type and control, and adjusting for it could bias the insurance coefficient) [7,8,11].

Table 3 presents hypothetical results from this study along with an explicit identification of the methodological problems that affect interpretation of each coefficient. These problems illustrate why the Table 2 fallacy is not merely an academic concern but has concrete implications for each variable in the model.

Taken together, these problems show that presenting a table of adjusted PRs and selecting those with *p* < 0.05 as “independent risk factors” is methodologically unjustifiable. Each coefficient poses different interpretation challenges, and the model as a whole cannot answer the implicit causal question (“what should we intervene on?”) without a causal framework.

If the researcher wishes to add value using that dataset, the analysis should be reframed according to the actual question. If the aim is prediction, the study should develop or validate a prediction model and assess discrimination, calibration, and validation. If the aim is causality, the exposure, target estimand, time zero, causal assumptions, and adjustment strategy should be specified. If the aim is local service planning, descriptive or operational epidemiology may be sufficient. If the aim is hypothesis generation, results should be reported explicitly as model-conditional associations without causal language.

## 11. Practical Recommendations

**Figure 1:**
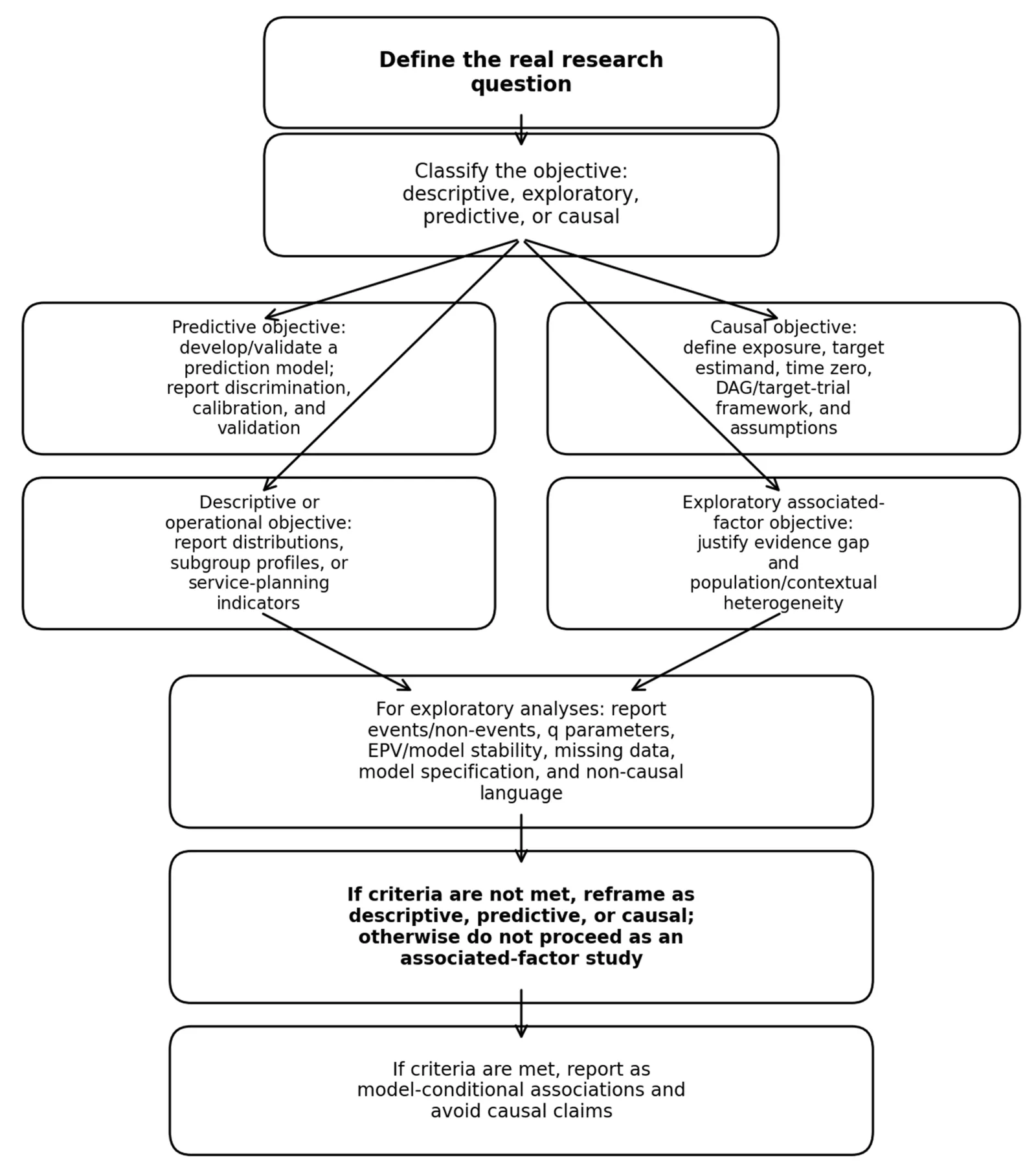
Decision algorithm to determine whether conducting an associated-factor study is appropriate.

Based on the discussion, practical recommendations should be framed as a single decision pathway rather than as separate rules for investigators, reviewers, and editors. An associated-factor study should pass three sequential checks: objective alignment, evidentiary contribution, and modeling credibility. Figure 1 summarizes this decision process as a practical algorithm.

First, authors should classify the real research question. If the interest is causal, the study should define an exposure, target estimand, time zero, causal assumptions, and adjustment strategy. If the interest is prediction, the study should be developed or validated as a prediction model with discrimination, calibration, and validation assessment. If the interest is local description or service planning, descriptive or operational epidemiology may be the appropriate design.

Second, an exploratory associated-factor analysis is justified only when there is a credible evidence gap, a plausible reason to expect population or contextual heterogeneity, or an explicit hypothesis-generating objective. The novelty criterion should not be satisfied by geography alone; authors should explain why the new setting could change interpretation or why prior evidence cannot be transported to the target population.

Third, authors should report model complexity and stability: number of events and non-events, q parameters or effective degrees of freedom, EPV or another sample-size justification, model specification, missing-data handling, and any validation or sensitivity analysis. Coefficients should be described as model-conditional associations or adjusted associations within the specified model, avoiding terms such as “effect,” “protective,” “determinant,” or “independent risk factor.”

Reviewers and editors can operationalize “insufficient novelty” by asking whether the manuscript identifies a substantive evidence gap, a plausible transportability or external-validity issue, a novel exposure or outcome context, or a planned exploratory objective. If none of these conditions is met, the manuscript should be reframed as descriptive, predictive, or causal according to the actual question, or its scientific contribution should be considered limited. These criteria complement existing reporting principles for observational studies, such as STROBE [39]. A practical decision and minimum-reporting checklist for associated-factor studies is presented in Table 4.

Beyond causal language and EPV, authors should report additional design and modeling features that affect the credibility of associated-factor analyses: missing-data mechanisms and handling, measurement error in key variables, selection processes in hospital-based or registry-based samples, clustering in multicenter data, sparse-data problems or separation in logistic models, multiplicity, and whether model selection occurred after inspecting the data [40,41]. These issues should not be treated as generic limitations appended at the end of the article, but as part of the minimum information required to interpret the fitted model.

## 12. Conclusions

Associated-factor studies can be useful when their objective is explicitly exploratory or descriptive, when there is a genuine evidence gap, and when there is a plausible reason why the population, exposure, or context may generate new information. Their contribution is limited when they merely reproduce well-established associations, are conducted because data are available, or use causal language that the design cannot support.

The central requirement is alignment between question, method, and interpretation. Exploratory models estimate model-conditional associations, not causal effects or validated predictions. Therefore, authors should define the research purpose, justify variable selection and model complexity, report events and effective parameters, avoid causal terminology, and acknowledge the limitations of interpreting multiple adjusted coefficients from a single model.

For authors, reviewers, and editors, the practical question is not whether multivariable models should be prohibited but whether a given associated-factor analysis answers a clear scientific question and adds information beyond what is already known. When that standard is met, these studies can generate useful hypotheses; when it is not, the analysis should be reframed as descriptive, predictive, or causal according to the actual objective.

## Figures and Tables

**Table 1:** Appropriate and inappropriate language for describing results from exploratory associated-factor models.

Inappropriate (Implies Causality)	Appropriate (Describes Association)
“X increases the risk of Y”	“X was associated with a higher prevalence of Y within the specified model”
“X is an independent risk factor for Y”	“X showed a model-conditional association with Y”
“X has a protective effect against Y”	“X was inversely associated with Y within the fitted model”
“X predisposes to developing Y”	“Participants with X had a higher frequency of Y”
“X contributes to the development of Y”	“An association between X and Y was observed in the adjusted model”
“The impact of X on Y was significant”	“The estimated association was [estimate], with a 95% confidence interval of [CI], and should be interpreted as a model-conditional association rather than a causal effect”
“X is a determinant of Y”	“X was one of the variables associated with Y within the specified model”
“The effect of X remained after adjustment”	“The model-conditional association for X remained after adjustment”

Note: Phrases in the left column imply that modifying X would change Y, which requires causal evidence that an exploratory model cannot provide. Phrases in the right column describe observed patterns without implying a causal mechanism.

**Table 2:** Factors associated with neurologic complications among hospitalized patients with an emerging tropical disease (n = 380, 45 events). Hypothetical data for illustrative purposes.

Variable	Crude PR	Adjusted PR	95% CI	*p* Value
Age, per 10 years	1.18	1.12	0.94–1.33	0.203
Male sex	1.31	1.24	0.78–1.97	0.364
Log10 viral load on admission	1.89	1.72	1.28–2.31	<0.001
Prior neurologic comorbidity	2.45	2.18	1.34–3.54	0.002
Days since symptom onset, per day	1.08	1.05	0.96–1.15	0.287

PR: prevalence ratio; CI: confidence interval. Poisson regression with robust variance, adjusted for all variables shown.

**Table 3:** Hypothetical results of an associated-factor study of poor glycemic control (HbA1c > 7%) in 1200 patients with type 2 diabetes, with identification of specific methodological issues.

Variable	Adjusted PR (95% CI)	*p* Value	Issue Type	Explanation
Years since diagnosis, per 5 years	1.38 (1.22–1.56)	<0.001	Temporality	May reflect accumulated disease history rather than a modifiable causal factor.
Treatment adherence	0.35 (0.26–0.47)	<0.001	Reverse causality/measurement error	Self-report may be affected by current control, health literacy, motivation, or access [8].
Insulin therapy	1.67 (1.22–2.29)	0.001	Confounding by indication	May indicate greater disease severity or prior treatment failure; not a harmful effect of insulin [15].
Microvascular complications	1.52 (1.14–2.03)	0.004	Ambiguous temporality	May be consequences of prior poor control rather than causes of current poor control [8].
Medical visits ≥ 4/year	0.82 (0.62–1.08)	0.156	Selection/indication	May reflect access, severity, or monitoring intensity.
Higher education	0.79 (0.59–1.06)	0.118	Overadjustment	Adjustment for access or adherence may attenuate or distort the association [9].
Low socioeconomic status	1.28 (0.96–1.71)	0.089	Residual confounding	Socioeconomic position may be imperfectly measured and mediated through care pathways.
BMI ≥ 30 kg/m^2^	1.18 (0.90–1.55)	0.224	Model structure/overadjustment	Adjustment for treatment may block or open pathways involving obesity, severity, and therapy assignment [7,9].

Note: Hypothetical data for illustrative purposes. The model simultaneously adjusted for all variables, generating the interpretation problems noted. CI: confidence interval; BMI: body mass index; PR: prevalence ratio.

**Table 4:** Decision and minimum-reporting checklist for associated-factor studies.

Domain	Minimum Item to Report	Why It Matters
Research purpose	State whether the analysis is descriptive, exploratory, predictive, or causal.	Prevents conflation of objectives.
Evidence gap	Explain why the analysis can add new information.	Avoids redundant associated-factor studies.
Population/context	Justify why associations may differ in this population or setting.	Addresses external validity and transportability.
Target parameter	Define whether coefficients are conditional associations, prediction parameters, or causal estimands.	Prevents causal overinterpretation.
Variable selection	Explain whether variables were selected a priori and why.	Reduces post hoc model construction.
Model complexity	Report events, non-events, q parameters/effective degrees of freedom, and EPV or another sample-size justification.	Allows assessment of stability and overfitting risk.
Model specification	Report functional forms, categories, interactions, clustering, and transformations.	Prevents hidden modeling decisions.
Missing data	Report extent, mechanism assumptions, and handling strategy.	Avoids complete-case bias.
Bias risks	Address measurement error, selection bias, sparse data/separation, multiplicity, and post hoc selection.	Makes limitations specific rather than ceremonial.
Interpretation language	Use “model-conditional association” or “adjusted association within the specified model.”	Avoids causal language.
Validation/sensitivity	Report internal validation, bootstrap stability, penalization, or sensitivity analyses when applicable.	Supports credibility of exploratory findings.
Transparency	State whether the analysis plan, code, or analytic decisions are available.	Improves reproducibility.

Note: This checklist is intended to complement, not replace, reporting frameworks for observational studies such as STROBE. It focuses on issues that are particularly common in associated-factor analyses.

## Data Availability

No new data were created or analyzed in this study. Data sharing is not applicable to this article.
